# Spatial-Temporal Distribution of Allelopathic Rice Roots in Paddy Soil and Its Impact on Weed-Suppressive Activity at the Seedling Stages

**DOI:** 10.3389/fpls.2022.940218

**Published:** 2022-07-05

**Authors:** Jiayu Li, Shunxian Lin, Huayan Ma, Yanping Wang, Haibin He, Changxun Fang

**Affiliations:** ^1^Fujian Provincial Key Laboratory of Agroecological Processing and Safety Monitoring, College of Life Sciences, Fujian Agriculture and Forestry University, Fuzhou, China; ^2^Key Laboratory of Crop Ecology and Molecular Physiology, Fujian Agriculture and Forestry University, Fuzhou, China

**Keywords:** rice (*Oryza sativa*), root distribution, allelopathy, rice allelochemical, benzoic acid derivatives, cinnamic acid derivatives

## Abstract

**Background:**

Allelochemicals secreted by allelopathic rice roots are transmitted to the receptor rhizosphere through the soil medium to inhibit the growth of the surrounding weeds. This research aimed to explore the relationships between the spatial-temporal distribution of rice roots in soil and weed-suppression ability at its seedling stage.

**Results:**

This study first examined the root distribution of three rice cultivars in paddy soil in both vertical and horizontal directions at 3–6 leaf stage. Then, an experiment using rice–barnyardgrass mixed culture was conducted to analyze the allelopathic potential and allelochemical content secreted by rice roots in different lateral soil layers. The results showed that allelopathic rice had a smaller root diameter and larger root length density, root surface area density, and root dry weight density than those of non-allelopathic rice, in the top 5 cm at 5- and 6-leaf stages. In particular, there were significant differences in root distribution at the horizontal distance of 6–12 cm. Besides, allelopathic rice significantly inhibited the above-ground growth of barnyardgrass co-cultured at 12 cm lateral distance *in situ*, and the content of benzoic acid derivatives in allelopathic rice in a 6–12 cm soil circle was higher than that observed at 0–6 cm distance. Moreover, correlation analysis confirmed that the distribution of roots in the horizontal distance was significantly correlated with weed inhibition effect and allelochemical content.

**Conclusion:**

These results implied that spatial distribution of allelopathic rice roots in paddy soil, particularly at the lateral distance, appears to have important impact on its weed-suppressive activity at the seedling stage, suggesting that modifying root distribution in soil may be a novel method to strengthen the ability of rice seedlings to resist paddy weeds.

## Introduction

Allelopathy refers to a plant (including microorganisms) that releases chemicals into the surrounding environment that have a direct or indirect effect on another plant (Rice, 1984). Allelochemicals, derived from bioactive secondary metabolites released by allelopathic rice, can inhibit the growth and development of associated weeds to a certain extent without the introduction of synthetic compounds, and are widely considered as a sustainable approach of weed control by many scientists (Rice, 1984; Olofsdotter et al., 2002; Li et al., 2015, 2019; Zhang et al., 2018a, 2019b; Serra et al., 2021). Rice allelopathy is mainly manifested in the 3-6 leaf stage (Li et al., 2015; Zhang et al., 2020), and International Rice Research Institute pointed out in the Rice Planting Manual that weed control within 30 days of rice transplantation had little impact on rice yield (Vergara, 1992).

Dilday et al. (2000) observed that allelopathic rice varieties PI312777 and PI338046 had larger root biomass than that of non-allelopathic rice varieties Lemont and M-201. These findings, however, did not immediately attract the attention of researchers. In 2013, Gealy et al. (2013) used ^13^C isotopes to study the differences in the root distribution of 11 rice cultivars in a field test in Arkansas. Results show that the allelopathic cultivars typically produced a greater fraction of their total root mass near the surface at 0–5 cm of soil depth when compared to the breeding selections or the non-suppressive cultivars, which tended to distribute their roots more evenly throughout the soil profile. These findings raised an interesting prospect for the first time that root proliferation near the soil surface might enhance the weed-suppressive activity of allelochemical exudates released from roots. All the above-mentioned findings aroused great interest in us. In 2019, we studied the differences in the morphological traits of roots in different potential allelopathic rice cultivars at the seedling stage in a hydroponic system (Li et al., 2019), and the results showed that allelopathic rice cultivars had significantly higher root lengths with thinner diameters, more number of root tips, and greater root biomass, which were significantly positively correlated with allelopathic inhibition. These results seem to indicate that the root system of allelopathic rice does have some relationship with the allelopathic activity of rice. We hypothesized that the rice varieties with a different spatial-temporal distribution of roots show different allelopathic activity in the field soil, particularly in the horizontal direction of the soil layer, which may be related to its inhibitory effect on the growth of adjacent weeds.

The root system plays a very important role in the agro-ecosystems and provides information about soil and crops to promote appropriate adaptive responses. The function of the plant root system is closely related to root morphology and physiological characteristics, and often adapts to environmental changes through morphological changes and temporal and spatial distribution (Wu and Cheng, 2014; Novoplansky, 2019). The growth and development of the root system and its spatial and temporal distribution in the soil can determine the absorption of water and nutrients during the growth process of crops (Fan et al., 2016; Hu et al., 2020), and also directly affect the growth and final yield of above-ground crops (Forde and Lorenzo, 2001; Zhang et al., 2009; Jeong et al., 2013). The root system of rice has strong plasticity and can improve drought avoidance and optimize the distribution of limited water resources by increasing the deep rooting of the crop systems (Uga et al., 2013; Zhang et al., 2019a). There are no reports on whether the root system of allelopathic rice influences the root distribution characteristics and allelopathic metabolism of roots in the horizontal soil, and thus exerts an inhibitory effect on the surrounding weeds.

Our previous study designed an inhibitory-circle method that can reduce the relative contribution of plant–plant competition to the maximum possible extent (He et al., 2012; Li et al., 2015). In this method, rice accession and barnyardgrass were cultured together in the paddy soil under natural conditions. The highest allelopathic activity of allelopathic rice accession PI312777 was at the 5-leaf stage, and the suitable distance of rice seedlings and barnyardgrass was 12 cm apart in a circle, which might be related to the spatial and temporal distribution of allelopathic rice roots through our conjecture. Therefore, the objectives of this study were to (1) evaluate the spatial-temporal distribution patterns of allelopathic rice roots in paddy soil in both horizontal and vertical directions at different seedling stages, (2) compare the spatial distribution difference of phenolic acid content, an important class of rice allelochemicals secreted by rice roots, and (3) analyze the correlations among root parameters, the phenolic acid content in the soil, and weed-suppressive activity.

## Materials and Methods

### Materials and Experimental Design

Two internationally recognized allelopathic rice cultivars, “PI312777” (PI) and “Taichung Native1” (TN), and one non-allelopathic rice cultivar, “Lemont” (Le) (Dilday et al., 2000), were used in this study. Mature barnyardgrass (*Echinochloa crus-galli*) seeds were collected from rice fields the year prior to the experiment and stored in a refrigerator for over 1 year.

The soil was collected randomly at a paddy field from the experimental site. The soil is typical silt loam with a pH of 6.42, organic matter of 258.1 mg kg^−1^, and a fertility status with a total N of 505.9 mg kg^−1^, available P of 51.2 mg kg^−1^, available K of 116.2 mg kg^−1^, and EC_25_ of 1.2 dS m^−1^. Soil samples were air-dried, mixed, and then sieved (2 mm mesh) to remove residual plant roots and branches to perform a series of experiments as described in the following section.

Two experiments were conducted in a greenhouse with 23–38°C night and daytime temperatures and 40–80% relative humidity maintained during the growing season in 2020. The seeds of rice varieties (PI, TN, and Le) and barnyardgrass were soaked in water for 24 h and pre-germinated in the sand.

Experiment 1 was conducted to evaluate the spatial-temporal distribution characteristics of the roots of allelopathic (PI and TN) and non-allelopathic (Le) rice cultivars. A total of 48 plastic pots (30 × 15 cm) containing 12 kg of soil described earlier were used for the experiment. Five pre-germinated rice seeds were spaced uniformly in the central area (1 cm diameter) of a pot, which was watered at a particular time every day to maintain soil moisture, and paddy weeds were removed manually during the experimental period. The experiments were conducted in a completely randomized design with four replicates for each cultivar at each leaf stage. When the rice seedlings were at the 3-, 4-, 5-, and 6-leaf stages, the seedlings were harvested, and their roots were collected for analysis as described in section Root Collection and Measurements.

Experiment 2 was conducted to determine allelopathic activity and temporal distribution difference of phenolic acid contents in allelopathic and non-allelopathic rice cultivars in soil. In order to intuitively and effectively observe the inhibitory potential of different cultivars on weeds, the established inhibitory-circle method was adopted for the study based on the results of experiment 1. A total of 32 plastic pots (30 cm diameter × 15 cm height) containing 12 kg of soil described above were used for the experiment. As shown in Supplementary Figure 1, five pre-germinated rice seeds were sown in the central area (1 cm diameter) of a pot, and water was added daily to the pot to maintain soil moisture. Paddy weeds were manually removed during the experimental period. When the rice seedlings grew to the 5-leaf stage, five germinated seeds of barnyardgrass were planted uniformly in a circle around the rice seedlings at 12 cm apart from the base of rice seedlings. The monoculture of five germinated barnyardgrass seeds without rice seedlings was set up as the control group. The experiments were conducted in a completely randomized design with eight replicates for each cultivar. At the 6-leaf stage, the roots were sampled from four randomly selected plastic pots of each rice cultivar as described in section Root Collection and Measurements. For the rest of the plastic pots of each rice cultivar, all barnyardgrass plants were harvested for allelopathic activity measurement as described in section Allelopathic Activity Measurement, and the soil of different plots was sampled for phenolic acid content analysis as described in section Quantification of Rice Allelochemicals in Different Soil Layers.

### Samples and Measurements

#### Root Collection and Measurements

In the first experiment, when the rice seedlings grew to the 3-, 4-, 5- and 6-leaf stages, respectively, the above-ground parts of allelopathic and non-allelopathic rice cultivars were cut. The soil with rice roots was cut into 0–5 cm and 5–10 cm depth circles, and root samples of these two depth plots were collected in horizontal distance intervals of 0–3, 3–6, 6–9, 9–12, and 12–15 cm with the base of rice seedlings as the center as shown in Supplementary Figure 2.

In the second experiment, according to the results of experiment 1, root samples of different rice cultivars at the 6-leaf stage were sampled in the 0–5 cm soil depth as described above and collected in horizontal distance intervals of 0–6 and 6–12 cm with the base of rice seedlings as the center as shown in Supplementary Figure 3.

All soil samples with rice roots in the different parts of experiments 1 and 2 obtained above were soaked in tap water in the laboratory and then washed with a metal-sieve stack (pore size of 0.40 mm), and all roots were manually collected. Clean roots were immediately scanned by an Epson Expression 11000XL scanner (Seiko Epson Co., Nagano-ken, Japan) to yield a grayscale image. Root length (cm), root surface area (cm^2^), and average root diameter (RD, mm) were determined using the WinRHIZO software (Regent Instruments Inc., Quebec, Canada). After the analysis, root samples were oven-dried at 105°C for 30 min and at 80°C for 48 h to measure the root dry weight (Li et al., 2019). Then, we calculated root length density (RLD), root surface area density (RSD), and root dry weight density (RWD) in different soil layers. RLD, RSD, and RWD were calculated as follows: *RLD* = *L*/*V* (cm cm^−3^), *RSD* = *S*/*V* (cm^2^ cm^−3^), and *RWD* = *W*/*V* (mg cm^−3^), where *L* is the root length, *S* is the root surface area, *W* is the root dry weight in each soil layer, and *V* is the volume of the core soil sampled in each layer (Jiang et al., 2016; Hu et al., 2020).

#### Allelopathic Activity Measurement

When the rice seedlings grew to the 6-leaf stages in the second experiment, all barnyardgrass samples co-cultured with allelopathic and non-allelopathic rice cultivars were harvested, and the plant height was measured. The above-ground parts of barnyardgrass were then cut and plant fresh weight was measured, and the parts were later oven-dried at 120°C for 30 min and at 80°C for 48 h to obtain their dry weight. To assess the allelopathic activity of rice cultivars, the inhibition percentage was calculated as follows: inhibition % = (1-T/C) × 100, based on the plant height, fresh weight, and dry weight of barnyardgrass co-cultured with three rice cultivars (T) and monoculture barnyardgrass in controls (C) (Li et al., 2015).

#### Quantification of Rice Allelochemicals in Different Soil Layers

The soil samples collected from experiment 2 at the 0–5 cm depth were taken at horizontal distance intervals of 0–6 and 6–12 cm with the base of rice seedlings as the center, as shown in Supplementary Figure 3. The samples from the same rice cultivar were mixed evenly, slightly air-dried, and sieved (<5 mm) after removing the plant fragments, and prepared for further extraction of phenolic acids.

The method for the extraction of phenolic acids from the soil, which are important rice allelochemicals, was based on the protocol proposed by Li et al. (2020) with slight modifications. Four replicates of soil (100 g) samples were collected, respectively, as described above and then placed into 250 mL conical flasks. Then, 100 mL of 0.25 mol L^−1^ of sodium citrate (pH 7.0) was added to the flask, which was then shaken for 2.5 h and centrifuged at 11,000 rpm for 10 min. The combined supernatant was filtered using a 0.45-μm filter for the quantitative analysis of phenolic acids.

The content of single phenolic acids in different soil layers was quantified using the external standard method by the solid-phase extraction and high-performance liquid chromatography (HPLC) as described previously (Li et al., 2019) with some modifications. The pH of the sample solutions acquired from each experiment was adjusted to 4.00 by adding phosphoric acid, and then NaCl was added to the solution to reach 8% (g/mL), which was filtered through a 0.45-μm membrane. The resulting filtrate was then loaded onto Cleanert PEP soild-phase extraction cartridges (Agela, China). The cartridge was eluted with water and then methanol, and the methanol fraction was concentrated with N_2_, which was resolved by 500 μL chromatographic methanol for quantitative analysis using HPLC. All samples were analyzed using an HPLC instrument (Waters e2695, Waters, USA) equipped with SunFireTW-C_18_ column (4.6 × 250 mm ID, 5 μm). The mobile phase was the mixture of methanol (A) and 1% phosphoric acid (B), and the gradient elution program was as follows: A/B (27/73, v/v), 10 min; A/B (50/50, v/v), 10 min; and A/B (73/27, v/v), 5 min. The mobile phase was eluted at a flow rate of 1.3 mL/min and detected at 280 nm. The injection volume was 10 μL and the column temperature was 30°C. Eight phenolic acids (protocatechuic, *p*-hydroxybenzoic, vanillic, syringic, *p*-coumaric, ferulic, salicylic, and cinnamic acid) were chosen as standards for the calibration curve. The concentration of single phenolic acids in each soil sample was quantified by interpolating the peak area on the HPLC chromatogram to a standard curve constructed from the peak area of the authentic phenolic acids (Li et al., 2020).

### Statistical Methods

SPSS 22.0 was used for the statistical analysis of the data. Data were presented as mean ± standard error (SE) values from three replicates for each experiment or determination. Variance in the mean values of root parameters, allelopathic activity, and phenolic acid contents among the different rice cultivars was analyzed by two-way ANOVA using SPSS (Version 20, Chicago, IL, USA), followed by Tukey's honestly significant difference (HSD) test at *p* < 0.05. The correlations between weed-suppressive activity and phenolic acid contents and root parameters (*n* = 15) were analyzed by using the “Corrplot” package in R (Wei and Simko, 2017).

## Results

### Spatial-Temporal Distribution Patterns of Allelopathic Rice Roots in Paddy Soil

#### Temporal Distribution of Root

**Figure 1:**
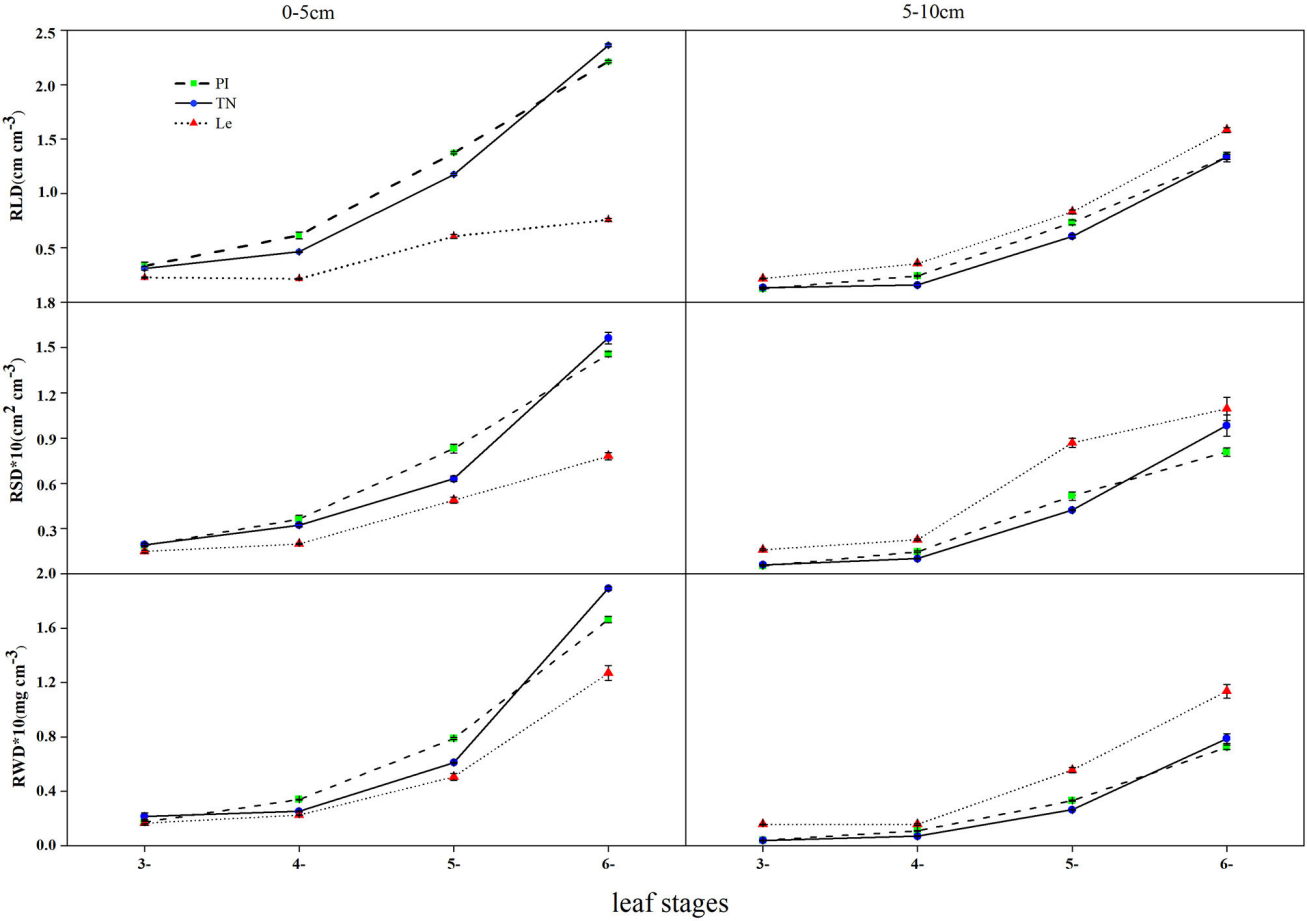
Root length density, root surface area density, and root dry weight density in the 0–5 and 5–10 cm soil layers at the 3–6 leaf stage. PI, allelopathic rice PI312777; TN, allelopathic rice Taichung Native1, and Le, non-allelopathic rice Lemont. Error bars indicate standard deviation.

Root length density (RLD), root surface area density (RSD), and root dry weight density (RWD) of three rice cultivars showed the same trend at the seedling stages (Figure 1). In the 0–5 and 5–10 cm soil layers, RLD, RSD, and RWD of allelopathic rice PI and TN and non-allelopathic rice Le increased with leaf stages, reaching a maximum at the 6-leaf stage.

Root length density differed significantly between allelopathic cultivars PI and TN and non-allelopathic rice Le, except that no significant difference was observed at the 3-leaf stage. In the soil depth of 0–5 cm, the RLD of allelopathic rice PI and TN was significantly higher than non-allelopathic rice Le (RLD = 2.213, 2.362, and 0.756 cm cm^−3^ for cultivars PI, TN, and Le, respectively) at the 6-leaf stage, while there were no significant differences between PI and TN. However, non-allelopathic rice had higher RLD than two allelopathic cultivars at the 6-leaf stage (RLD = 1.335, 1.326, and 1.583 cm cm^−3^ for cultivars PI, TN, and Le, respectively) in the 5–10 cm soil layers.

Similar trends for RSD and RWD were observed, except for no significant difference in RWD between allelopathic and non-allelopathic rice cultivars at the 4-leaf stage. At the soil depth of 0–5 cm, RSD and RWD of allelopathic rice PI and TN were significantly higher than those of non-allelopathic rice Le (RSD = 0.146, 0.156, and 0.078 cm^2^ cm^−3^ for cultivars PI, TN, and Le, respectively; RWD = 0.166, 0.189, and 0.127 mg cm^−3^ for cultivars PI, TN, and Le, respectively) at the 6-leaf stage.

#### Vertical Distribution of Root

Root length density (RLD) of three rice cultivars declined with increasing soil depth in the 0–10 cm soil layer at 3–6 leaf stages (Figure 1). Regardless of the leaf stages, the surface soil layer had a higher density of roots for allelopathic rice PI and TN. At the 6-leaf stage, the RLD number of PI and TN in 0-5 cm soil layer accounted for 62% and 64% of the total roots, respectively. However, RLD of non-allelopathic rice Le was higher in the 5–10 cm soil depth at different seedling stages, constituting 67% of the total root at the 6-leaf stage.

The root surface area density (RSD) and dry weight density (RWD) followed a similar trend in the 0–10 cm soil layer at 3–6 leaf stages (Figure 1). PI and TN had significantly higher RSD and RWD than Le in the 0-5 cm soil layer (*p* < 0.05). Approximately, 74 and 75% of root biomass of PI and TN (range: 69–81% and 70–85% for PI and TN, respectively) were concentrated in the upper soil (0-5 cm) during the seedling stages, which was significantly higher than Le, except for the 3-leaf stage. However, RSD and RWD values of Le in the 5–10 cm soil horizon were significantly higher than those observed in PI or TN.

#### Horizontal Distribution of Roots at Different Distances

**Figure 2:**
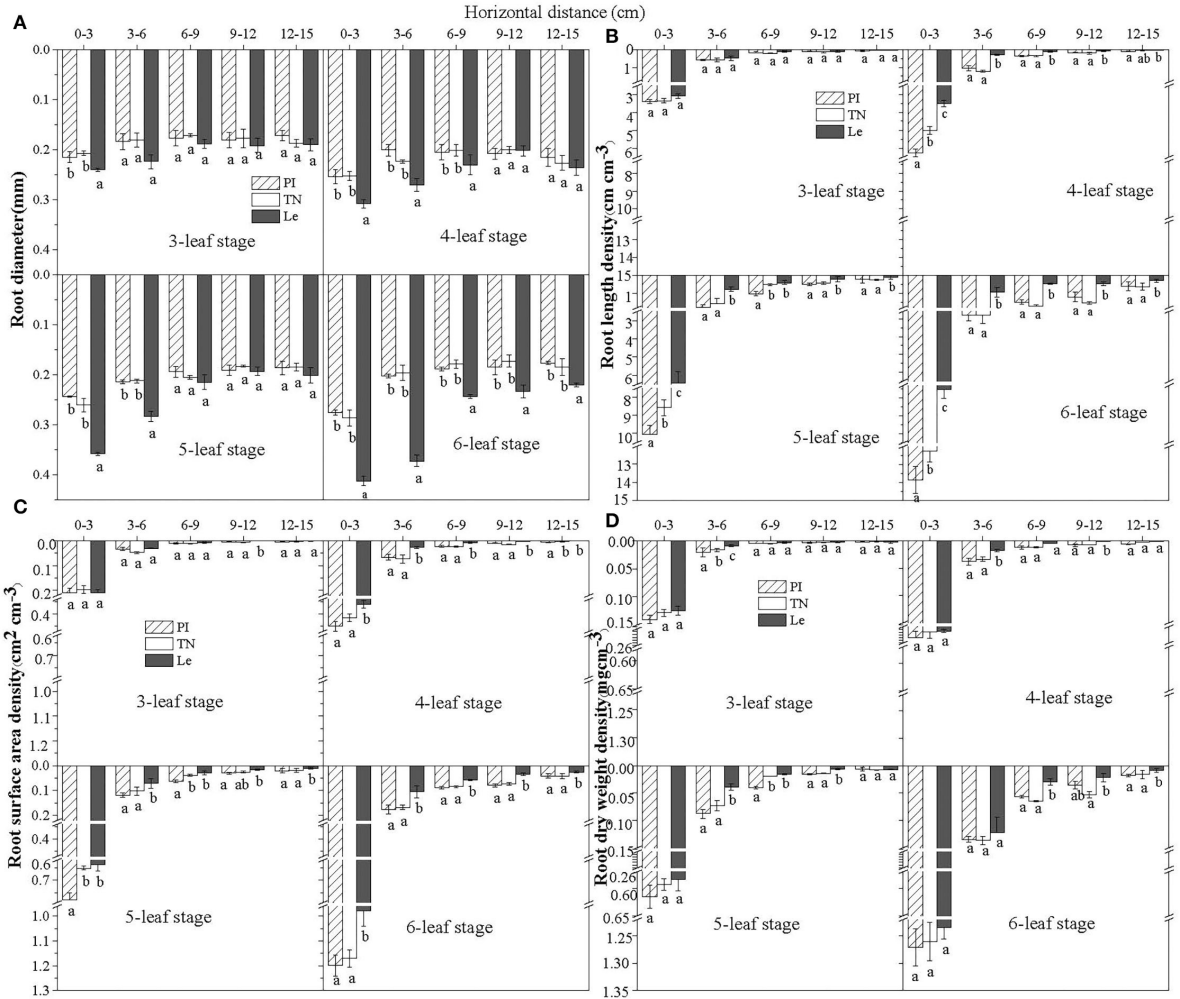
Root diameter **(A)**, root length density **(B)**, root surface area density **(C)**, and root dry weight density **(D)** of three rice cultivars at different horizontal distances from the base of rice seedlings in the 0–5 cm soil depth at the seedling stages. PI: allelopathic rice PI312777, TN: allelopathic rice Taichung Native1, and Le: non-allelopathic rice Lemont. Error bars indicate standard deviation. Significant differences (*p* < 0.05) between rice cultivars at the same distance were indicated by different lowercase letters, according to Tukey's honestly significant difference test.

The root diameter (RD) of three rice cultivars declined with increasing soil horizontal distance in the 0–5 cm soil depth at the seedling stages (Figure 2A). The roots at 0–3 cm horizontal distance from rice seedlings had the largest RD, followed by the roots collected from 3 to 6 cm distance. At distances > 6 cm, RD values of non-allelopathic rice Le at 6–9, 9–12, and 12–15 cm were significantly higher than that of allelopathic rice PI and TN at the 6-leaf stage (*P* < 0.05).

The distribution patterns in root length density (RLD) of three rice cultivars at different horizontal distances at the seedling stages is shown in Figure 2B. There was a trend toward increasing RLD with increasing leaf stages within each soil layer. At the 6-leaf stage, RLD at 0–3 cm distance was significantly higher for PI than observed in either TN or Le (RLD =13.9, 12.3, and 7.5 cm cm^−3^ for PI, TN, and Le, respectively). RLD was lower at distances > 3 cm from the base of rice seedlings; however, the distribution pattern was similar to that observed for the distance of 0–3 cm soil horizon. RLD of PI decreased from 2.8 cm cm^−3^ at 3–6 cm to 1.5 cm cm^−3^ at 6–9 cm and 1.2 cm cm^−3^ at 9–12 cm to 0.6 cm cm^−3^ at 12–15 cm distances from seedlings. At 4-, 5-, and 6-leaf stages, there were no significant differences between the RLD at 6–9 cm and 9–12 cm, but RLD of allelopathic rice was significantly higher than that of non-allelopathic rice (*p* < 0.05).

The root surface area density (RSD) and root length density (RLD) followed a similar trend at different horizontal distances from the base of rice seedlings in the 0–5 cm soil depth at the seedling stages (Figure 2C). At the 3-leaf stage, there were no significant differences in RSD with each distance among the three rice cultivars. However, RSD was significantly lower in Le than in PI or TN, and there was no significant difference between PI and TN. At the 6-leaf stage, RSD of allelopathic rice PI at 6–9 and 9–12 cm showed no significant differences, which was, however, significantly higher than RSD at 12–15 cm (RSD = 0.18, 0.09, 0.08, and 0.04 cm^2^ cm^−3^ at 3–6, 6–9, 9–12, and 12–15 cm, respectively)

Patterns of root dry weight density (RWD) of three rice cultivars at different horizontal distances at the seedling stages are shown in Figure 2D. Although allelopathic rice had higher RWD at 0–3 cm distance than non-allelopathic rice, there was no significant difference. RWD of PI declined from 0.13 mg cm^−3^ at 3–6 cm to 0.05 mg cm^−3^ at 6–9 cm and 0.04 mg cm^−3^ at 9–12 cm to 0.01 mg cm^−3^ at 12–15 cm distance from seedlings. Although there were no significant differences between RWD values at 6–9 cm and 9–12 cm for three rice cultivars, the RWD of allelopathic rice was significantly higher than that of non-allelopathic rice (*p* < 0.05).

Based on the above results, the root characteristics of allelopathic and non-allelopathic rice showed the most significant difference at the 6-leaf stage. According to the vertical distribution of roots, the roots of allelopathic rice were concentrated mainly in the top 0–5 cm soil layer, and their contents quickly decreased with depth, while highly significant differences were observed in the root measures of allelopathic rice at 0–5 cm depth. From the horizontal distribution of roots, the roots of seedling rice were mainly distributed in the circle layer of 6 cm from the growth center, while the soil at the lateral distance > 12 cm had few roots. No significant differences were observed in all four root measures between soil layers at distances 6–9 and 9–12 cm, but the roots of allelopathic rice were significantly higher than those of non-allelopathic rice. Therefore, to simplify the analysis, the sampled root lateral region in 0–5 cm top soil was divided into two circles (0–6 and 6–12 cm) to further study the effects of root distribution on rice allelopathy at the seedling stages in experiment 2.

### Effects of Root Distribution in Soil on Rice Allelopathy at the Seedling Stages

#### Allelopathic Activity of Rice Toward Barnyardgrass *In situ*

**Figure 3:**
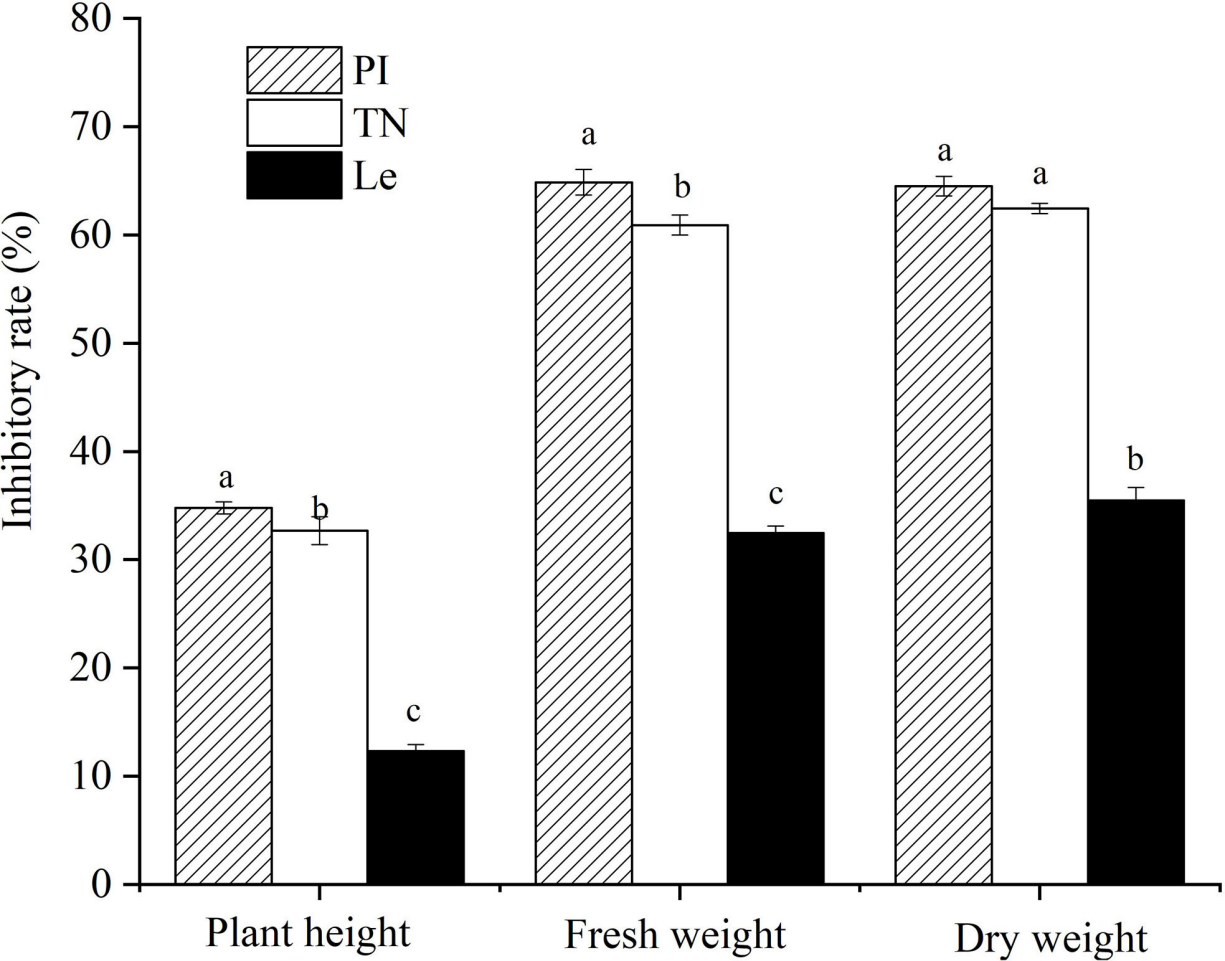
Effects of three rice cultivars on barnyardgrass *in situ*. PI: allelopathic rice PI312777, TN: allelopathic rice Taichung Native1, and Le: non-allelopathic rice Lemont. Error bars indicate standard deviation. Significant differences (*p* < 0.05) between rice cultivars were indicated by different lowercase letters, according to Tukey's honestly significant difference test.

Compared with barnyardgrass monocultures, the inhibitory rates on plant height, fresh weight, and dry weight of barnyardgrass at a 12 cm distance were 34.8, 64.9, and 64.5%, respectively, in PI, 32.7, 60.9, and 62.4%, respectively, in TN, and 12.3, 32.5, and 35.5% in Le, respectively (Figure 3). The results showed that two allelopathic rice cultivars highly suppressed the above-ground growth of barnyardgrass co-cultures at a 12 cm distance from rice seedlings, other than non-allelopathic rice.

#### Lateral Distribution of Rice Roots in Two Soil Circles

In order to explore whether the above results (Figure 3) were related to the lateral distribution of rice roots, RLD, RSD, and RWD for two soil layers (0–6 and 6–12 cm circles) were averaged and analyzed. One-way ANOVA showed significant effects of rice cultivars and lateral distance (Table 1) and significant cultivars × lateral distance interaction (*p* < 0.05). The simple-effect analysis further showed significant differences in all the three root measures (*p* < 0.05) between 0–6 and 6–12 cm lateral distance, regardless of rice cultivars. Highly significant differences were observed in three root measures (*p* < 0.05) between allelopathic rice PI or TN and non-allelopathic rice Le at 0–6 cm soil circle. At a distance of 6–12 cm, no significant differences were observed in RLD and RSD between PI and TN, but the root measures of allelopathic rice were significantly higher than those of non-allelopathic rice.

**Table 1:** Effects of different rice cultivars and lateral distance on root distribution.

		**RLD (cm cm^**−3**^)**	**RSD (cm^**2**^ cm^**−3**^)**	**RWD (mg cm^**−3**^)**
0–6 cm	PI	5.560 ± 0.120a	0.432 ± 0.005a	0.461 ± 0.055a
	TN	5.409 ± 0.479a	0.457 ± 0.043a	0.480 ± 0.033a
	Le	2.590 ± 0.114b	0.324 ± 0.007b	0.347 ± 0.021b
6–12 cm	PI	1.402 ± 0.060a	0.083 ± 0.003a	0.047 ± 0.004a
	TN	1.627 ± 0.043a	0.089 ± 0.004a	0.057 ± 0.006a
	Le	0.479 ± 0.100b	0.036 ± 0.003b	0.023 ± 0.001b
ANOVA		*P*-Value		
Cultivars		0.001	0.001	0.001
Lateral distance		0.001	0.001	0.001
Cultivars × lateral distance		0.001	0.007	0.018

*Means ± standard error (SE) from three replications for each determination is shown. Significant differences (p < 0.05) between rice cultivars at the same distance were indicated by different lowercase letters, according to Tukey's honestly significant difference test*.

#### Lateral Distribution of Rice Allelochemicals in Two Soil Circles

The inhibition of weeds by allelopathic rice mainly results from the production of rice allelochemicals. We detected the presence of eight phenolic acids (protocatechuic, *p*-hydroxybenzoic, vanillic, syringic, *p*-coumaric, ferulic, salicylic, and cinnamic acid), which are recognized as rice allelochemicals. The results showed that variation in the content of each phenolic acid in the three rice cultivars was inconsistent with the increase in lateral distance (Table 2). The reported phenolic acid compounds with allelopathic potential are mainly classified into benzoic acid derivatives and cinnamic acid derivatives. Based on the chemical structure, the sum of the contents of five phenolic acids (protocatechuic, *p*-hydroxybenzoic, vanillic, syringic, and salicylic acid) is termed as benzoic acid derivatives content, and the sum of the remaining three phenolic acids (cinnamic, *p*-coumaric, and ferulic acid) is defined as cinnamic acid derivatives content.

**Table 2:** The content of eight phenolic acids in two soil circles of different allelopathic rice varieties.

**Phenolic acid**	**Distance**	**Content of phenolic acids (ng g**^**−1**^ **soil)**		
		**PI**	**TN**	**Le**
Protocatechuic acid	0–6 cm	1.35 ± 0.11	1.87 ± 0.01	1.58 ± 0.03
	6–12 cm	2.54 ± 0.06	2.52 ± 0.04	1.58 ± 0.04
*p-*Hydroxybenzoic acid	0–6 cm	32.49 ± 0.69	36.62 ± 0.23	47.85 ± 0.50
	6–12 cm	82.02 ± 0.37	84.43 ± 0.19	40.18 ± 0.75
Vanillic acid	0–6 cm	18.17 ± 0.55	14.91 ± 0.03	6.06 ± 0.04
	6–12 cm	36.53 ± 0.13	34.52 ± 0.19	6.34 ± 0.05
Syringic acid	0–6 cm	2.18 ± 0.19	1.40 ± 0.07	1.47 ± 0.16
	6–12 cm	3.83 ± 0.09	3.26 ± 0.06	1.24 ± 0.05
*p*-Coumaric acid	0–6 cm	14.37 ± 0.24	17.78 ± 0.73	10.94 ± 0.71
	6–12 cm	54.96 ± 0.42	52.21 ± 0.67	15.35 ± 0.53
Ferulic acid	0–6 cm	1.24 ± 0.16	0.88 ± 0.02	ND
	6–12 cm	2.05 ± 0.73	1.85 ± 0.53	ND
Salicylic acid	0–6 cm	3.02 ± 0.06	2.17 ± 0.12	5.72 ± 0.21
	6–12 cm	11.10 ± 0.12	12.17 ± 0.12	8.30 ± 2.80
Cinnamic acid	0–6 cm	1.75 ± 0.33	0.56 ± 0.04	0.69 ± 0.15
	6–12 cm	0.58 ± 0.04	0.56 ± 0.04	0.54 ± 0.02

*Means±standard error (SE) from three replications for each determination is shown. “ND” means compounds not detected in the analysis. PI and TN, allelopathic cultivars PI312777 and Taichung Native1; Le, non-allelopathic cultivar Lemont*.

**Figure 4:**
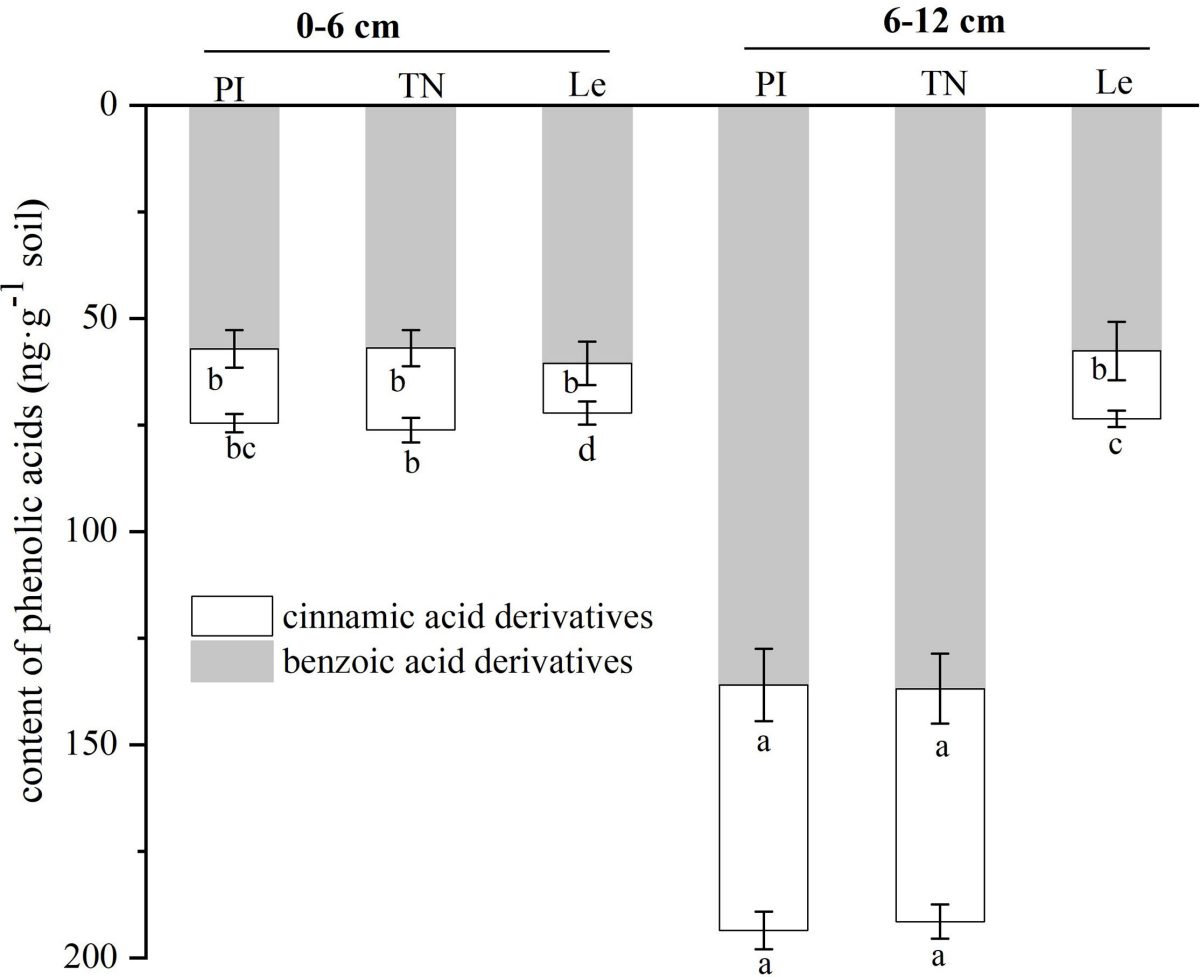
Contents of benzoic acid derivatives and cinnamic acid derivatives in two soil circles of three rice cultivars. PI: allelopathic rice PI312777, TN: allelopathic rice Taichung Native1, and Le: non-allelopathic rice Lemont. Error bars indicate standard deviation. Significant differences (*p* < 0.05) between rice cultivars were indicated by different lowercase letters, according to Tukey's honestly significant difference test.

As shown in Figure 4, the contents of both types of phenolic acids in the 6–12 cm soil circle were higher than those observed in the 0–6 cm soil circle in allelopathic rice, while the cinnamic acid derivative contents of non-allelopathic rice were slightly higher in the 6–12 cm soil layer. At the same time, although there was no significant difference in the benzoic acid derivatives content of the three rice cultivars at 0–6 cm lateral distance, the contents of two kinds of phenolic acids in the 6–12 cm soil layer of allelopathic rice were significantly higher than those of non-allelopathic rice. In addition, the total amount of all phenolic acids showed a similar trend at two different horizontal distances from the base of three rice seedlings.

#### Correlation Between Root Distribution and Weed-Suppressive Activity and the Content of Rice Allelochemicals in Soil

**Figure 5:**
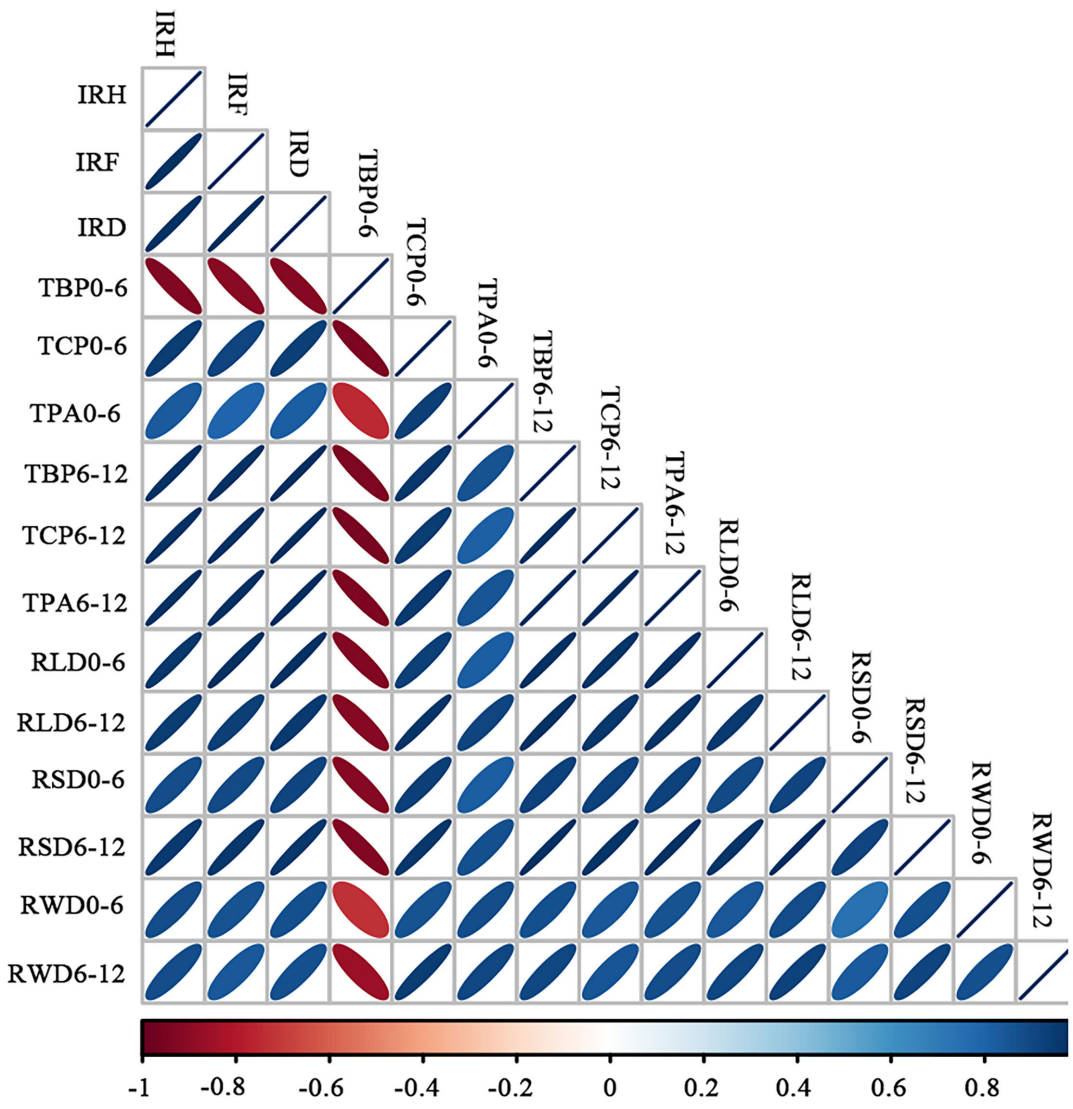
Correlation plot between allelopathic activity, rice allelochemicals in soil, and root parameters at a lateral distance. IRH, rice allelopathic activity (% inhibition on plant height of barnyardgrass); IRF, rice allelopathic activity (% inhibition on fresh weight of barnyardgrass); IRD, rice allelopathic activity (% inhibition on the dry weight of barnyardgrass); TBP, the content of benzoic acid derivatives in soil; TCP, the content of cinnamic acid derivatives in the soil; TPA, total phenolic acid content in soil; RLD, root length density; RSD, root surface area density; and RWD, root dry weight density. The gradient of the legend is a function of the strength of the correlation; while the slope of the ellipse indicates a negative or positive correlation (i.e., toward the left is a positive correlation and toward the right is a negative correlation). The shape of the ellipse indicates the strength of the correlation; a diffuse shape indicates a weak correlation.

We studied the correlations between weed-suppressive activity (% inhibition on plant height, fresh weight, and dry weight of barnyardgrass), rice allelochemicals in soil (the contents of benzoic acid derivatives, cinnamic acid derivatives, and total phenolic acids), and root parameters (RLD, RSD, and RWD) at two lateral distances (Figure 5). Overall, weed-suppressive activity and rice allelochemicals in the soil had positive correlations with RLD, RSD, and RWD at both distances, except for the content of benzoic acid derivatives in soil at 0–6 cm. At a 6–12 cm horizontal distance from the base of rice seedlings, weed-suppressive activity (IRH, IRF, and IRD) had a stronger correlation with the contents of allelochemicals (TBP, TCP, and TPA) and root measures (*p* < 0.05). Besides, RLD and RSD values at 6–12 cm were highly correlated with the contents of benzoic acid derivatives (TBP), cinnamic acid derivatives (TCP), and total phenolic acid (TPA) in soil (*p* < 0.05). These two root parameters simultaneously had a stronger correlation with the inhibition rate of rice on plant height (IRH), fresh weight (IRF), and dry weight (IRD) of barnyardgrass (*p* < 0.05).

## Discussion

Roots play a vital role in connecting the plant to the soil, which can synthesize, accumulate, and secrete a diverse array of compounds referred to as root exudates that significantly impact the soil environment (Laliberté, 2017; Tsunoda and Dam, 2017). Root distribution in the soil (how many root segments of individual roots exist in each soil compartment) is the most crucial factor in determining the release of root exudates into the soil (Tajima, 2021). Allelochemicals secreted by the roots of allelopathic rice are transmitted to the receptor rhizosphere through the soil medium to inhibit the growth of the surrounding weeds (Li et al., 2020; Xu et al., 2021). In this study, it was found that the lateral distribution of roots in the soil of allelopathic rice at the seedling stage was related to its weed-suppressive effect *in situ*, which provided a new method for improving the inhibitory ability of allelopathic rice at the seedling stage and supplied a scientific basis for the application strategy of expanding the weed-suppressive range in the field by strengthening the root traits of rice cultivars.

Our hypothesis was that the spatial-temporal distribution of rice cultivars with different allelopathic activity in the underground soil may be different, which proved to be true for root length density (RLD), root surface area density (RSD), and root dry weight density (RWD) of three rice cultivars at 5- and 6-leaf stages (Figure 1), the leaf stages showing the strongest allelopathic activity (Li et al., 2015; Zhang et al., 2020). From the vertical direction of the soil layer, the roots of allelopathic rice were mainly concentrated in the upper layer of 0–5 cm, while the roots of non-allelopathic rice were mainly distributed in the soil at 5–10 cm depth (Figure 1), which was consistent with the experimental results of Gealy and Moldenhauer (2012) and Gealy et al. (2013). From the horizontal direction of the soil layer at 0–5 cm depth, the RLD and RSD values of allelopathic rice roots in each sampled soil layer were higher than those of non-allelopathic rice, particularly at the 5-leaf or 6-leaf stage (Figures 2B,C). However, the difference in the RWD values between the circle layers (0–3 and 3–6 cm) around the growth center was not significant (Figure 2D), which may be explained by the results that allelopathic rice has a small root diameter but long roots, while non-allelopathic rice has large root diameter but short roots (Figure 2A). When comparing our results to those of previous studies (Li et al., 2019), it must be pointed out that the root surface area of PI and Le showed no significant difference at the seedling stage in a hydroponic system. These contradictory results may be a consequence of the differences in the planting medium. RLD and RSD in crop plants play an important role in improving water and nutrient use efficiency in soil, and also play a unique role in the interaction between root exudates and microorganisms in the soil (van Dam and Bouwmeester, 2016; Hu et al., 2020). Data generated in this study revealed that the roots of allelopathic rice mainly proliferated in the upper soil profile, extending to the lateral direction as far as possible, while those of non-allelopathic rice mostly grew in the deeper soil layer. It was expounded that the main functions of the roots of two kinds of rice cultivars may be different in different soil varieties.

Soil is the natural substrate where roots grow, and therefore it is of more practical significance to explore the regulation of rice allelopathy by the characteristics of the rice root system in the underground soil. In order to simulate the actual rice planting conditions in the field, the study adopted the inhibitory-circle method established earlier (Li et al., 2015), whereby the rice was mixed with barnyardgrass in paddy soils at equal distances, resulting in the formation of weed-suppressive circles (Supplementary Figure 1). Based on the results of the horizontal distribution of rice roots in the current study, few roots were distributed at the lateral distance > 12 cm from the base of rice seedlings (Figures 2B–D). Meanwhile, the weed-suppressive effect is a combination of rice allelopathy and interspecific competition (Kong et al., 2008; Xu et al., 2021). Therefore, it was considered that the 6–12 cm distance between the rice seedlings and barnyardgrass was a suitable distance to reduce plant–plant interference from the competition as much as possible. Several studies have shown that roots of allelopathic rice always grew toward the roots of weeds (intrusive pattern), while the roots of all weeds tended to avoid growing toward the roots of rice (Zhang et al., 2018b; Xu et al., 2021), which implied that the roots of barnyardgrass at 6–12 cm horizontal distance might be negligible with the help of manual selection. Similar to Li et al. (2020), we found that allelopathic rice cultivars significantly inhibited the above-ground growth of barnyardgrass co-cultures at a 12 cm lateral distance *in situ* (Figure 3).

Rice allelopathy is the result of an interaction effect between rice allelochemicals, including phenolic acids, and other allelochemicals, known as the “allelochemicals cocktail” (Li et al., 2020). The eight phenolic acids selected in our study are widely recognized as rice allelochemicals (Seal et al., 2004; Li et al., 2019, 2020; Zhang et al., 2019b, 2020). Usually, a single compound could not manifest the allelopathic effect of rice exudates on the target plants. In this paper, the sum of contents of eight phenolic acids in root exudates (Table 2) was expressed as the total phenolic acid contents in the root exudates of three rice cultivars (Figure 4). Recent studies have indicated that the allelopathic potential of benzoic acid derivatives, one kind of phenolic acid, was higher than that of cinnamic acid derivatives in a laboratory bioassay (Reigosa and Malvido, 2007; Li et al., 2020). This study highlighted that the contents of benzoic acid derivatives in the 6–12 cm soil circle were higher than those observed in the 0–6 cm soil circle in allelopathic rice, while that of non-allelopathic rice showed no significant difference (Figure 4), similar to other previous studies (Li et al., 2020; Zhang et al., 2020). The difference in the number of allelochemicals in soil layers at lateral distances of allelopathic rice may also be partly driven by soil microbial composition, because the rhizosphere microbiota could impact the transport of root exudate metabolites from rice to weed in soil, such as the adsorption and desorption of benzoic acid derivatives and cinnamic acid derivatives with soil matrix (Tharayil et al., 2006; Sun et al., 2014). In the follow-up study, we will further explore whether the distribution of allelopathy rice in the lateral soil can regulate the distribution of the rhizosphere microflora in soil and inhibit the adjacent weeds.

The correlation analysis confirmed that root distribution at the horizontal distance was significantly correlated with the allelopathic activity on the growth of barnyardgrass *in situ* and allelochemical contents secreted by root in the horizontal soil (Figure 5). These results implied that the spatial-temporal distribution of allelopathic rice roots in the paddy soil may have important impact on rice allelopathy at the seedling stages. Weston and Mathesius (2013) reported some flavonoids can accumulate at the root tip of white clover and were exuded into the soil from root cap cells, mediating allelopathic interactions in the plant rhizosphere. Although the current study did not accurately locate the release location of phenolic acid from rice roots as performed by Zhu et al. (2016), it is well-known that allelopathic rice mainly secretes phenolic acid allelochemicals through its roots (Li et al., 2019; Zhang et al., 2019b, 2020). Therefore, we can conclude that the distribution of allelopathic rice roots in the lateral distance was bound to affect the action distance of allelopathic substances secreted by roots, thus affecting their inhibitory effect on weeds. It is suggested that the growth of rice roots in the soil would be used as a regulation target for rice allelopathy. Several reports have shown that many plant hormones could regulate the growth and development of rice roots, particularly brassinolide, ethylene, and abscisic acid (Zhao et al., 2015; Cai et al., 2018). Consequently, more in-depth work is needed to identify inducing agents for effectively improving the allelopathic potential of traditional or improved rice cultivars, via screening for regulators of root distribution.

## Conclusion

Enhancing the weed-suppressive activity of rice at its seedling stage may be an important strategy to apply rice allelopathy for controlling paddy weeds. This study showed that allelopathic rice cultivars had small root diameter and larger root length density, root surface area density, and root dry weight density in the top 5 cm of the vertical soil profile at 5- and 6-leaf stages. In particular, there were significant differences in root distribution at the horizontal distance of 6–12 cm between allelopathic rice and non-allelopathic rice. Furthermore, these root traits had strong correlations with the phenolic acid content at the lateral 6–12 cm of the soil layer and weed-suppressive activity *in situ*, suggesting that strengthening root traits or modifying the distribution of rice roots in soil may be a novel method to enhance the ability of rice seedlings to resist paddy weeds. However, additional studies involving the mechanism of regulating rice–weed interactions by the spatial distribution of rice roots in paddy soil are required.

## Data Availability Statement

The original contributions presented in the study are included in the article/Supplementary Material, further inquiries can be directed to the corresponding authors.

## Author Contributions

Material preparation, data collection, and analysis were performed by JL, SL, and HM. The first draft of the manuscript was written by JL. All authors commented on the previous versions of the manuscript and contributed to the study's conception, and design. All authors read and approved the final manuscript.

## Funding

This work was supported by the National Natural Science Foundation of China (31871556, 31701802) and the Natural Science Foundation of Fujian Province (2020J01546).

## Conflict of Interest

The authors declare that the research was conducted in the absence of any commercial or financial relationships that could be construed as a potential conflict of interest.

## Publisher's Note

All claims expressed in this article are solely those of the authors and do not necessarily represent those of their affiliated organizations, or those of the publisher, the editors and the reviewers. Any product that may be evaluated in this article, or claim that may be made by its manufacturer, is not guaranteed or endorsed by the publisher.

## References

[B1] Cai Z. P. Yang W. Zhang H. Y. Luo J. J. Wu F. H. Jing X.. (2018). Ethylene participates in the brassinolide-regulated asymmetric growth of *O. sativa* root. S. Afr. J. Bot. 119, 86–93. 10.1016/j.sajb.2018.08.017

[B2] Dilday R. H. Mattice J. D. Moldenhauer K. A. (2000). An overview of rice allelopathy in the USA, in Rice Allelopathy. International Rice Research Instituteeds Kim K. U. Shin D. H. (Manila), 15–26.

[B3] Fan J. L. McConkey B. Wang H. Janzen H. (2016). Root distribution by depth for temperate agricultural crops. Field Crop Res. 189, 68–74. 10.1016/j.fcr.2016.02.01325306104

[B4] Forde B. Lorenzo H. (2001). The nutritional control of root development. Plant Soil 232, 51–68. 10.1023/A:1010329902165

[B5] Gealy D. R. Moldenhauer K. Duke S. (2013). Root distribution and potential interactions between allelopathic Rice, Sprangletop (*Leptochloa spp*.), and barnyardgrass (*Echinochloa crus-galli*) based on ^13^C isotope discrimination analysis. J. Chem. Ecol. 39, 186–203. 10.1007/s10886-013-0246-7 23397455

[B6] Gealy D. R. Moldenhauer K. A. K. (2012). Use of ^13^C isotope discrimination analysis to quantify distribution of barnyardgrass and rice roots in a four-year study of weed-suppressive rice. Weed Sci. 60, 133–142. 10.1614/WS-D-10-00145.1

[B7] He H. B. Wang H. B. Fang C. X. Lin Z. H. Yu Z. M. Lin W. X. (2012). Separation of allelopathy from resource competition using rice/barnyardgrass mixed-cultures. PLoS ONE. 7, e37201. 10.1371/journal.pone.0037201 22590655 PMC3349635

[B8] Hu Y. J. Ma P. H. Wu S. F. Sun B. H. Feng H. Pan X. L.. (2020). Spatial-temporal distribution of winter wheat (*Triticum aestivum* L.) roots and water use efficiency under ridge-furrow dual mulching. Agr. Water Mange. 240, 106301. 10.1016/j.agwat.2020.106301

[B9] Jeong J. S. Kim Y. S. Redillas M. C. F. R. Jang G. Jung H. Bang S. W.. (2013). *OsNAC5* overexpression enlarges root diameter in rice plants leading to enhanced drought tolerance and increased grain yield in the field. Plant Biotechnol. J. 11, 101–114. 10.1111/pbi.12011 23094910

[B10] Jiang H. Bai Y. Y. Du H. Y. Hu Y. Rao Y. F. Chen C.. (2016). The spatial and seasonal variation characteristics of fine roots indifferent plant configuration modes in new reclamation saline soil of humid climate in China. Ecol. Eng. 86, 231–238. 10.1016/j.ecoleng.2015.11.020

[B11] Kong C. H. Hu F. Wang P. Wu J. L. (2008). Effect of allelopathic rice varieties combined with cultural management options on paddy field weeds. Pest. Manag. Sci. 64, 276–282. 10.1002/ps.1521 18172879

[B12] Laliberté E. (2017). Below-ground frontiers in trait-based plant ecology. New Phytol 213, 1597–1603. 10.1111/nph.14247 27735077

[B13] Li J. Y. Guo X. K. Zhang Q. Liu C. H. Lin Z. H. Yu Z. M.. (2015). A novel screening method for rice allelopathic potential: the inhibitory-circle method. Weed Res. 55, 441–448. 10.1111/wre.12166

[B14] Li J. Y. Lin S. X. Zhang Q. Li L. Hu W. W. He H. B. (2020). Phenolic acids and terpenoids in the soils of different weed-suppressive circles of allelopathic rice. Arch. Agron Soil Sci. 66, 266–278. 10.1080/03650340.2019.1610560

[B15] Li J. Y. Lin S. X. Zhang Q. X. Zhang Q. Hu W. W. He H. B. (2019). Fine-root traits of allelopathic rice at the seedling stage and their relationship with allelopathic potential. Peer J. 10.7717/peerj.7006 31223525 PMC6570997

[B16] Novoplansky A. (2019). What plant roots know? Semin. Cell Dev. Biol. 92, 126–133. 10.1016/j.semcdb.2019.03.009 30974171

[B17] Olofsdotter M. Jensen L. B. Courtois B. (2002). Improving crop competitive ability using allelopathy-an example from rice. Plant Breed. 121, 1–9. 10.1046/j.1439-0523.2002.00662.x

[B18] Reigosa M. J. Malvido E. P. (2007). Phytotoxic effects of 21 plant secondary metabolites on *Arabidopsis thaliana* germination and root growth. J. Chem. Ecol. 33, 1456–1466. 10.1007/s10886-007-9318-x 17577597

[B19] Rice E. L. (1984). Allelopathy, 2nd edn. Academic Press.

[B20] Seal A. N. Haig T. Pratley J. E. (2004). Evaluation of putative allelochemicals in rice root exudates for their role in the suppression of arrowhead root growth. J. Chem. Ecol. 30, 1663–1678. 10.1023/B:JOEC.0000042075.96379.71 15537166

[B21] Serra N. S. Shanmuganathan R. Becker C. (2021). Allelopathy in rice: a story of momilactones, kin recognition, and weed management. J. Exp. Bot. 72, 4022–4037. 10.1093/jxb/erab084 33647935

[B22] Sun B. Wang P. Kong C. H. (2014). Plant-soil feedback in the interference of allelopathic rice with barnyardgrass. Plant Soil 377, 309–321. 10.1007/s11104-013-2004-6

[B23] Tajima R. (2021). Importance of individual root traits to understand crop root system in agronomic and environmental contexts. Breed. Sci. 71, 13–19. 10.1270/jsbbs.20095 33762872 PMC7973490

[B24] Tharayil N. Bhowmik P. C. Xing B. S. (2006). Preferential sorption of phenolic phytotoxins to soil: implications for altering the availability of allelochemicals. J. Agr. Food Chem. 54, 3033–3040. 10.1021/jf053167q 16608227

[B25] Tsunoda T. Dam N. M. (2017). Root chemical traits and their roles in belowground biotic interactions. Pedobiologia 65, 58–67. 10.1016/j.pedobi.2017.05.00727145568

[B26] Uga Y. Sugimoto K. Ogawa S. Rane J. Ishitani M. Hara N.. (2013). Control of root system architecture by *DEEPER ROOTING 1* increases rice yield under drought conditions. Nat. Genet. 45, 1097-1102. 10.1038/ng.2725 23913002

[B27] van Dam N. M. Bouwmeester H. J. (2016). Metabolomics in the rhizosphere: tapping into belowground chemical communication. Trends Plant Sci. 21, 256–265. 10.1016/j.tplants.2016.01.008 26832948

[B28] Vergara B. S. (1992). A Farmer's Primer on Growing Rice, Revised ed. International Rice Research Institute, Manila.

[B29] Wei T. Simko V. (2017). R Package “Corrplot”: Visualization of a Correlation Matrix (version 0.84). Available online at: https://github.com/taiyun/corrplot (accessed July 15, 2021).

[B30] Weston L. A. Mathesius U. (2013). Flavonoids: their structure, biosynthesis and role in the rhizosphere, including allelopathy. J. Chem. Ecol. 39, 283–297. 10.1007/s10886-013-0248-5 23397456

[B31] Wu W. M. Cheng S. H. (2014). Root genetic research, an opportunity and challenge to rice improvement. Field Crops Res. 165, 111–124. 10.1016/j.fcr.2014.04.013

[B32] Xu Y. Cheng H. F. Kong C. H. Meiners S. (2021). Intraspecific kin recognition contributes to interspecific allelopathy: a case study of allelopathic rice interference with paddy weeds. Plant Cell Environ. 44, 3709–3721. 10.1111/pce.14083 33993534

[B33] Zhang A. Wang X. P. Luo Y. Z. Zhang L. Yao Y. Han L.. (2019a). *OsABA8ox2*, an ABA catabolic gene, suppresses root elongation of rice seedlings and contributes to drought response. Crop J. 8, 480–491. 10.1016/j.cj.2019.08.006

[B34] Zhang H. Xue Y. Wang Z. Yang J. C. Zhang J. H. (2009). Morphological and physiological traits of roots and their relationships with shoot growth in “super” rice. Field Crop Res. 113, 31–40. 10.1016/j.fcr.2009.04.00426566229

[B35] Zhang Q. Li L. Li J. Y. Wang H. B. Fang C. X. Yang X. Y.. (2018a). Increasing rice allelopathy by induction of barnyardgrass (*Echinochloa crus-galli*) root exudates. J. Plant Growth Regul. 37, 745–754. 10.1007/s00344-017-9770-y

[B36] Zhang Q. Zhang Q. X. Lin S. X. Wang P. Li J. Y. Wang H. B.. (2020). Dynamic analysis on weeds inhibition and phenolic acids of allelopathic rice in field test. Arch. Agron Soil Sci. 67, 1809–1821. 10.1080/03650340.2020.1811973

[B37] Zhang Q. Zheng X. Y. Lin S. X. Gu C. Z. Li L. Li J. Y.. (2019b). Transcriptome analysis reveals that barnyardgrass exudates increase the allelopathic potential of allelopathic and non-allelopathic rice (*Oryza sativa*) accessions. Rice. 12, 30. 10.1186/s12284-019-0290-1 31062105 PMC6502933

[B38] Zhang T. S. Fan B. Wang P. (2018b). Barnyardgrass root recognition behaviour for rice allelopathy. Agron. J. 8, 39. 10.3390/agronomy8040039

[B39] Zhao F. Y. Cai F. X. Gao H. J. Zhang S. Y. Wang K. Liu T.. (2015). ABA plays essential roles in regulating root growth by interacting with auxin and MAPK signaling pathways and cell-cycle machinery in rice seedlings. Plant Growth Regul. 75, 535–547. 10.1007/s10725-014-0017-7

[B40] Zhu X. C. Skoneczny D. Weidenhamer J. D. Mwendwa J. M. Weston P. A. Gurr G. M.. (2016). Identification and localization of bioactive naphthoquinones in the roots and rhizosphere of Paterson's curse (*Echium plantagineum*), a noxious invader. J. Exp. Bot. 67, 3777–3788. 10.1093/jxb/erw182 27194735 PMC4896362

