# Supplementary material for: Spatial-Temporal Distribution of Allelopathic Rice Roots in Paddy Soil and Its Impact on Weed-Suppressive Activity at the Seedling Stages
Source: Front Plant Sci. 2022 Jul 5;13:940218. doi: 10.3389/fpls.2022.940218 (PMC9294529; doi:10.3389/fpls.2022.940218)
Supplement: Supplementary file 1 [file Table_1.DOCX]

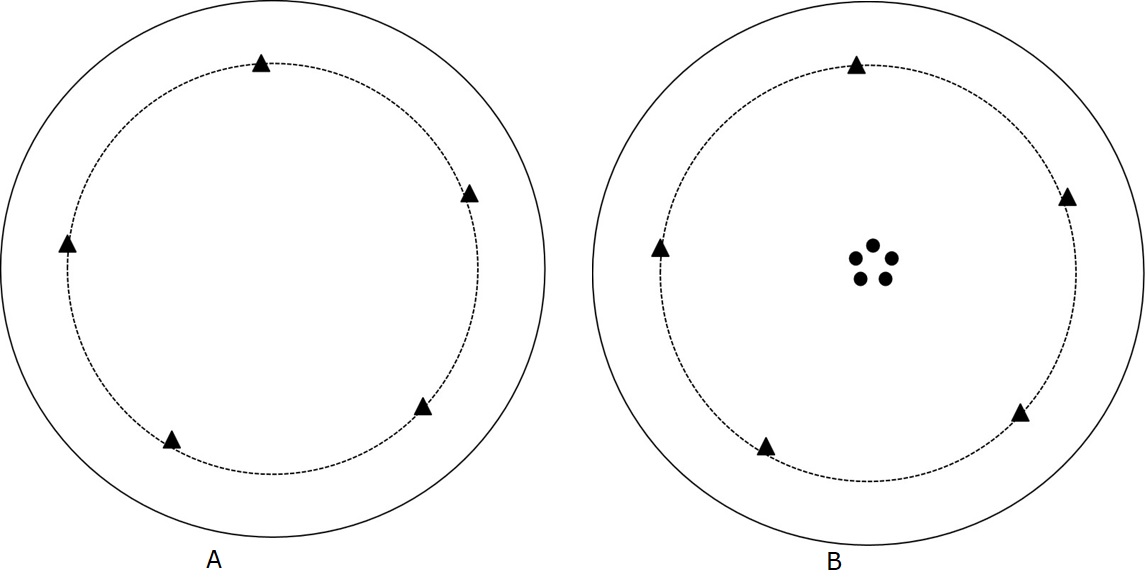


**Supplementary Figure 1.** The culture mode of pot experiment (A: control group; B: co-culture of rice and barnyardgrass), with black points representing rice planting areas and black triangle representing barnyardgrass planting areas.

**
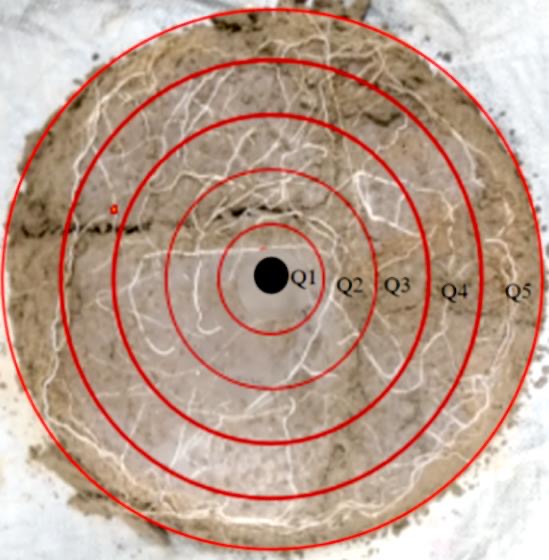
**

**Supplementary Figure 2.** Schematic diagram of rice root sampling in the horizontal direction, with black points representing rice planting areas and red circles representing different soil radii centered on the rice planting site in the first experiment, Q1: 0-3cm, Q2: 3-6cm, Q3: 6-9cm, Q4: 9-12cm, Q5: 12-15cm


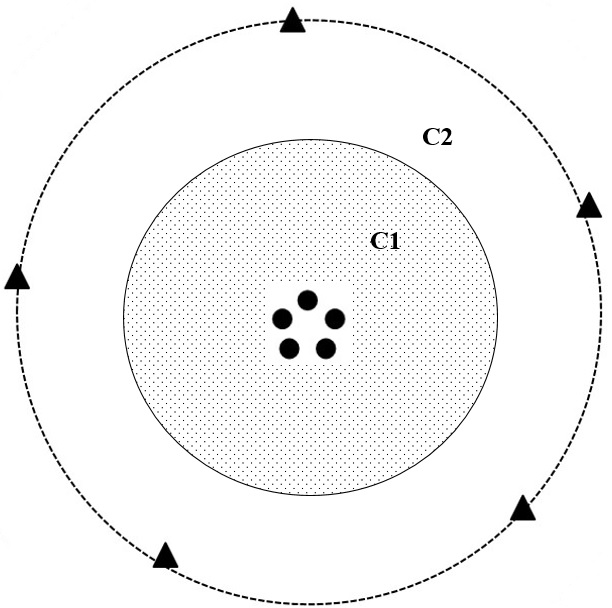


**Supplementary Figure 3.** Schematic diagram of rice root sampling on the horizontal direction (C1: 0-6cm, C2: 6-12cm), with black points representing rice planting areas and black triangle representing barnyardgrass planting areas.
